# Dr. C. L. Robertson on the Treatment of Insanity Resulting from Injury to the Head

**Published:** 1848-01-01

**Authors:** C. Lockhart Robertson

**Affiliations:** MILITARY ASYLUM, YARMOUTH


### ON THE APPLICATION OF THE TREPHINE TO THE TREATMENT OF INSANITY, THE RESULT OF INJURY TO THE HEAD.

BY C. LOCKHART ROBERTSON, M.D., MILITARY ASYLUM, YARMOUTH.

SEVERE injuries of the head, whether producing, in the first instance, concussion, or compression, are occasionally, though rarely, followed by permanent mental alienation. Thus, of 1220 cases, reported by Mons. Esquirol,* 18 or 1·4 per cent. only were caused by blows or falls on the head.

Every variety of insanity may result from this exciting cause. Thus, of 482 cases of melancholia† (Lympémanie ou Mélancholie), 10 or 2·0 per cent. were the result of injury to the head. Of 588 cases of mania,‡ 13 or 2·5 per cent. were caused in the same manner. Of 235 cases of dementia,§ 3 or 1·2 per cent. had the same origin.

Injuries of the head likewise produce moral insanity—*i. e.*, morbid perversion of the desires and affections (depending on disease of the moral principle or conscience, that power which conveys a certain conviction of what is right and wrong in regard to the exercise of the moral feelings) unattended by disorder of the intellectual faculties.

"There are instances," says Dr. Prichard,|| "in which a slight peculiarity of character, not amounting to insanity, has remained long, and perhaps through the life of the individual, who has sustained a severe injury of the head. Sometimes this constitutes a kind of moral insanity; the temper is more irritable, the feelings are less under restraint than previously."

Partial alteration of character is stated by Dr. Griesinger,¶ to be the occasional result of injury to the head. Case 559, at present in the asylum, is a good illustration of this remark. A fall from his horse produced alteration of character, attended by such unsettledness of purpose as to necessitate his confinement.

Injury to the head may act either as a predisposing or as an exciting cause of insanity. "Les chutes sur la tête,** même dès la première enfance, prédisposent à la folie, et en sont quelquefois la cause excitante." "A fall or blow may predispose to maniacal excitement."††

Many years may elapse between the receipt of the injury and the decided manifestation of the mental disorder. "Un enfant de trois ans fait une chute sur la tête ;‡‡ depuis il se plaint de céphalalgie ; à la puberté, le mal de tête augmente et la manie se déclare à l'âge de dix-

* "Des Maladies Mentales considérées sous les Rapports Médicales Hygiéniques et Médica-légales," tom. i. pp 62, 64. Paris, 1838.

† Esquirol, op. cit. tom. i. p. 435. ‡ Ib. tom. ii. p. 144. § Ib. p. 235.

|| "A Treatise on Insanity and other Disorders affecting the Mind," p. 202. London, 1835.

¶ "Die Pathologie und Therapie der Psychischen Krankeiten," seite 136. Stuttgart, 1845.

** Esquirol, op cit. tom. i. p. 68.

†† Dr Conolly, "Clinical Lectures on the Principal Forms of Insanity," delivered at the Middlesex Lunatic Asylum at Hanwell.—*Lancet*, Nov. 29, 1845.

‡‡ Esquirol, op. cit. tom. i. p. 68.

sept ans." "In some cases of slowly advancing insanity which I have met with, connected with general paralysis, there has been reason to suspect that a predisposing cause was a violent fall on the head some years previous to the appearance of the mental disorder."* "Blödsinn mit manie und dergl. stellen sich in andern Fällen viel später, 1, 2, 6, sogar 10 Jahre nach der Kopfverletzung ein."†

In all cases of insanity, the result of injury to the head, the *prognosis* will be very much influenced by the existence or non-existence of a depressed portion of skull. In the latter instance, we must be guided by the variety and extent of the mental alienation. In the former, a reasonable hope may be entertained that, by the removal of the predisposing or exciting cause—namely, the depressed portion of bone, the patient may be restored to the use of his faculties. In stating this opinion, I am fully aware that I differ from Dr. Conolly, who says‡ "that a depression existing even to a small extent, often appears to induce incurable insanity." I have not met with any other notice in works on insanity regarding the influence of a depressed portion of skull on mental alienation.

The following is a case of monomania, complicated, as this disease generally is, with moral insanity,|| and cured by the removal of the exciting cause.

*A case of Monomania caused by a depression in the Skull, and cured by the Operation of Trephine.*

No. 455, æt. 23, a sailor, was admitted into the Cumberland Lunatic Asylum, on the 10th of February, 1845.

Ten years prior, he fell from the mast of a ship, which accident was followed by an attack of acute mania.

In six weeks he recovered the use of his intellectual faculties, but continued so ungovernable in his temper and violent in his conduct, as to render him unfit to be at large, and to necessitate his removal to the Asylum.

On admission he complained of frequent pains in the part of the head on which he fell, and also entertained the delusion that these pains were caused by his mother beating him. Otherwise his intellectual faculties were sound. Various symptoms of disease of the moral principle were present. He was morose, taciturn, and insolent. He entertained an ungrounded dislike to his relations, and was subject to violent fits of passion.

After being some time in the asylum, his delusion gave way, and the intellectual powers of his mind remained sound; his conduct, however,

* Conolly, loc. cit. † Griesinger, loc. cit. ‡ Loc. cit.

|| I endeavoured, in the Northern Journal of Medicine for June 1846, to illustrate the fact, first pointed out by Dr. Prichard, that monomania (partial insanity), the most prominent symptom of which is the existence of a delusion or series of delusions, having reference to the individual so affected, is generally attended by perversion of the desires and affections (moral insanity). So that, though a monomaniac be capable of reasoning correctly on subjects unconnected with his delusion, he is unable to appreciate his moral responsibility, is unconscious of right and wrong, and, therefore, unfit to be at large, however harmless his delusion may apparently be.

continued ungovernable, and his language abusive; while kind words made no impression on his wayward temper. He still complained of pains in his injured part.

On examining his head, I discovered a very distinct depression on the posterior superior margin of the right parietal bone, the situation to which he referred the pains.

In consultation with my colleague, Mr. Furness, of Percy-street, Newcastle, consulting surgeon to the institution, it was decided that the depressed portion of skull be removed by the trephine.

On the 3d of January, 1846, the operation was skilfully performed by Mr. Furness. The patient bore it well, and the wound healed, without a bad symptom. The portion of the cranium removed was healthy in appearance on both of its surfaces. It adhered very firmly to the dura mater, requiring considerable force for its removal. It was altered considerably in form, appearing to have been indented, rather than fractured, which is not improbable, seeing the accident occurred to the patient when only thirteen years of age.

By the 1st of February his conduct was, and had been, since the operation, in every way improved. He had had no bursts of passion; answered civilly when spoken to, and was grateful for the relief afforded him. He looked forward with pleasure to his return home, which was promised to take place as soon as the weather improved. He had for the last fortnight been working in the farm, and stated that since the operation, he had been free from the pain in the head, from which he formerly suffered.

On the 20th of March he was discharged "cured," having, since the performance of the operation, shown no symptom of his previous malady.

Sir A. Cooper, in commenting on a case of Mr. Cline's, in which, by the operation of trephine, a man had been restored to health, who had passed thirteen months in "a state of perfect oblivion, deprived of all powers of mind, volition, or sensation," in consequence of a fall from the yard-arm, which had caused a slight depression on the head, says,* "It appears, therefore, that in cases of depression we should not be prevented from trephining, however distant the period may be at which the accident occurred; and the patient may, after any interval, be restored to the powers of body and mind." The case I have related corroborates this opinion of Cooper, as likewise does another case of Mr. Cline's, referred to by Dr. Wigan,† in which, the result of a blow with the end of a round roller,—a reprimand from a schoolmaster,—a state of hopeless idiocy had been induced. The trephine was applied as a last resource in a place where there appeared to be a slight depression. "We cannot do any harm," said Mr. Cline; "he must otherwise soon fall a sacrifice." From the upper surface of the portion of bone removed, projected a long spicula piercing the brain.

The boy entirely recovered the use of his intellect.

It would appear that injuries to the head, instead of producing insa-

* "Lectures on the Principles and Practice of Surgery," p. 135. London, 1835.

† "The Duality of the Mind," &c., p. 104. London, 1844.

nity, may even occasionally improve the mental powers. It is related by Dr. Cox,* that a son of the late Dr. Priestly was restored to reason from idiocy by a fall from a window.

A patient lately discharged "cured" from the Military Lunatic Asylum, had been admitted in a state of chronic mania (of some standing), his ideas being incoherent, his conversation rambling and unconnected, his memory, particularly of recent events, deficient. One morning he ran his head with such violence against the wall as to produce concussion of the brain. On recovering from this state, his ideas became gradually collected, his conversation coherent, and his memory returned. So entirely did he recover from his mental aberration as to be enabled to resume military duty, and to rejoin his regiment.

Dr. Cheyne states† "that, in consequence of external injuries of the head, the recollection of a language long forgotten has been restored."

"In other instances," says Dr. Prichard,‡ "there has been, after injury to the head, greater energy and activity, more of excitement in the general character, which has been thought a change for the better, rather than a morbid alteration."

"Cases of this description are sometimes very remarkable. I have been informed on good authority, that there was, some time since, a family, not far from this city, consisting of three boys who were all considered as idiots. One of them received a severe injury of the head: from that time his faculties began to brighten, and he is now a man of good talents, and practises as a barrister. His brothers are still idiotic or imbecile. Van Swieten§ mentions the case of a girl who was imbecile till she received an injury of the head, and underwent the application of a trephine for the removal of a depressed portion of skull: she recovered and became intelligent. Haller has reported the case of an idiot, whom a wound in the head restored to understanding.

A somewhat similar case is that of father Mobillon, who, says Dr. Cox,|| "acquired, after the operation of trepanning, a sudden increase of his intellectual faculties."

* "On Insanity," &c., p. 104.

† "Essays on Partial Derangement of the Mind in supposed connexion with Religion," p. 72. Dublin, 1843.

‡ Op. cit. p. 202.

§ Comment. in Boerhaavii Aphorismos, tom. i.

